# Metabolic Disruptions and Non-Communicable Disease Risks Associated with Long-Term Particulate Matter Exposure in Northern Thailand: An NMR-Based Metabolomics Study

**DOI:** 10.3390/biomedicines13030742

**Published:** 2025-03-18

**Authors:** Churdsak Jaikang, Giatgong Konguthaithip, Yutti Amornlertwatana, Narongchai Autsavapromporn, Sirichet Rattanachitthawat, Nitip Liampongsabuddhi, Tawachai Monum

**Affiliations:** 1 Department of Forensic Medicine, Faculty of Medicine, Chiang Mai University, Chiang Mai 50200, Thailand; churdsak.j@cmu.ac.th (C.J.); kongkiat.shang@gmail.com (G.K.); yutti.amornlert@cmu.ac.th (Y.A.); nitip.liam@cmu.ac.th (N.L.); 2 Metabolomics Research Group for Forensic Medicine and Toxicology, Department of Forensic Medicine, Faculty of Medicine, Chiang Mai University, Chiang Mai 50200, Thailand; 3 Division of Radiation Oncology, Department of Radiology, Faculty of Medicine, Chiang Mai University, Chiang Mai 50200, Thailand; narongchai.a@cmu.ac.th; 4 General Education Office, Faculty Agricultural Technology, Burapha University, Sakaeo 27160, Thailand; sirichet@buu.ac.th

**Keywords:** particulate matter, blood metabolomics, health effect, NMR-based metabolomics

## Abstract

**Background/Objectives**: Particulate matter (PM) is a primary health hazard associated with metabolic pathway disruption. Population characteristics, topography, sources, and PM components contribute to health impacts. **Methods**: In this study, NMR-based metabolomics was used to evaluate the health impacts of prolonged exposure to PM. Blood samples (*n* = 197) were collected from healthy volunteers in low- (control; CG) and high-exposure areas (exposure; EG) in Northern Thailand. Non-targeted metabolite analysis was performed using proton nuclear magnetic resonance spectroscopy (^1^H-NMR). **Results**: Compared to CG, EG showed significantly increased levels of dopamine, N6-methyladenosine, 3-hydroxyproline, 5-carboxylcytosine, and cytidine (*p* < 0.05), while biopterin, adenosine, L-Histidine, epinephrine, and norepinephrine were significantly higher in CG (*p* < 0.05). These metabolic disturbances suggest that chronic exposure to particulate matter (PM) impairs energy and amino acid metabolism while enhancing oxidative stress, potentially contributing to the onset of non-communicable diseases (NCDs) such as cancer and neurodegenerative conditions. **Conclusions**: This study highlighted the connection between sub-chronic PM2.5 exposure, metabolic disturbances, and an increased risk of non-communicable diseases (NCDs), stressing the critical need for effective PM2.5 reduction strategies in Northern Thailand.

## 1. Introduction

Particles in the air that have an aerodynamic dimension of 2.5 μm or less are known as fine particulate matter (PM2.5), and they are a serious health risk to humans because of their capacity to enter deep into the lungs [1]. These particles, composed of heavy metals, polycyclic aromatic hydrocarbons, and carbon compounds, originate from various sources, including combustion processes, industrial activities, and natural events [2]. The composition of PM2.5 varies based on geographic location, season [3], meteorological condition [4], and pollution source [5]. Changes in composition and sources influence their physicochemical properties and toxicological effects [6]. Given the diversity of PM2.5 components, their health effects can vary significantly based on exposure levels and particle composition.

Exposure to PM2.5 is a significant public health issue. In 2015, prolonged exposure to ambient PM2.5 was associated with around 4.2 million deaths globally, making it the fifth leading global risk factor and responsible for 7.6% of worldwide mortality [7]. The harmful health effects of PM2.5 exposure are primarily attributed to inflammation and oxidative stress [8,9], which contribute to a variety of diseases, including pulmonary, cardiovascular, neurodegenerative, and oncological conditions. Both short-term and long-term exposure to PM2.5 have been linked to increased morbidity and mortality, highlighting the urgent need to understand its health impacts [10,11].

Research on PM2.5 exposure has expanded to include metabolomics, a powerful tool for uncovering biochemical alterations associated with environmental exposures. To enhance the mechanistic understanding of PM2.5-induced health effects, recent studies have employed proton nuclear magnetic resonance (^1^H-NMR)-based metabolomics to investigate blood metabolic profiles in individuals exposed to ambient PM2.5. Plasma metabolome consists of biologically active compounds that reflect endogenous metabolic processes as well as exogenous environmental influences [12]. Metabolomic profiling has provided valuable insights into metabolic pathways associated with inflammation, oxidative stress, and other biochemical mechanisms linked to PM2.5 exposure [5,13]. Such approaches help elucidate the systemic impact of air pollution at a molecular level and may inform strategies for prevention and intervention.

PM2.5 exposure leads to alterations in lipid metabolism [14,15], amino acid pathways [16], and energy metabolism [17]. Lipidomic analyses have shown that PM2.5 exposure affects lipid profiles, including increased levels of oxidized phospholipids and disrupted lipid homeostasis [18], which are risk factors for cardiovascular diseases [19,20]. Additionally, amino acid metabolism, particularly branched-chain [21] and aromatic amino acids [22], has been linked to metabolic disorders in individuals exposed to PM2.5 [23]. Perturbation in these pathways suggests that PM2.5 exposure not only induces acute inflammatory responses [24] but also contributes to long-term metabolic imbalances that could increase susceptibility to chronic diseases [25,26].

Despite the growth of research on PM2.5-related health risks, uncertainties remain regarding the specific components, sources, and contributions of PM2.5 to different health outcomes. The toxic effects of PM2.5 may differ depending on particle composition, which varies by region and pollution sources [27]. For example, PM2.5 originating from vehicular emissions may have a different toxicological impact compared to PM2.5 from biomass burning [28]. Thus, identifying the major contributing sources and their respective health effects is crucial for developing targeted public health interventions and regulatory policies.

To address existing knowledge gaps, this study investigates the long-term impact of PM2.5 exposure by analyzing whole blood metabolomes using ^1^H-NMR-based techniques. By comparing individuals from regions with high and low levels of PM2.5 exposure in Thailand, this study seeks to elucidate the distinct contributions of various pollution sources and geographic factors to metabolic alterations. This approach will provide a comprehensive understanding of the impact of PM2.5 exposure on metabolic pathways and its potential role in disease pathogenesis.

Furthermore, integrating metabolomic data with environmental monitoring will offer novel insights into the molecular mechanisms underlying PM2.5 toxicity. The findings of this study will contribute to a more in-depth understanding of PM2.5-related health effects and inform the development of targeted public health interventions. Given the increasing burden of air pollution-related diseases, identifying metabolic biomarkers associated with PM2.5 exposure may serve as a valuable tool for early disease detection and risk assessment. Additionally, elucidating the biochemical perturbations induced by PM2.5 exposure can support future research on mitigating its adverse effects through pharmacological or lifestyle interventions. Ultimately, this research has the potential to inform public health policies aimed at reducing the health burden of PM2.5 exposure and improving global air quality standards.

## 2. Materials and Methods

### 2.1. Chemical Reagents

Analytical-grade chemical reagents were all utilized. We purchased deuterium oxide (D_2_O) and trimethylsilyl propanoic acid (TSP) from Sigma-Aldrich (St. Louis, MO, USA). We bought acetonitrile from Merck (Darmstadt, Germany).

### 2.2. Participants and Study Design

RAD-2564-08613, Research ID: 8613, was authorized by the Chiang Mai University Faculty of Medicine’s Ethics Committee. Mae Jam District in Chiang Mai Province represented the exposure group (high PM2.5 exposure), whereas Wattananakorn District in Sakeaw Province served as the control group (low PM2.5 exposure). Participants were recruited from these two regions of Thailand. In total, 99 people were in the exposure group and 98 were in the control group out of the 197 healthy volunteers who took part in the study. Heparinized tubes were used to draw about 5 mL of blood from each participant in October 2022. The samples were then kept at −80 °C until they could be examined further.

### 2.3. Preparation of Blood Samples

Whole blood samples were processed for NMR analysis using a modified version from Somtua and her colleagues [29]. Briefly, acetonitrile was used to extract blood samples, and each sample was subsequently mixed with 0.6 mL of 0.1 M TSP dissolved in D_2_O. To reduce the signal from water protons, ^1^H-NMR spectra were obtained on a 500 MHz spectrometer with water suppression.

### 2.4. ^1^H-NMR Spectroscopy

A Bruker AVANCE 500 MHz spectrometer (Bruker, Bremen, Germany), fitted with a Carr–Purcell–Meiboom–Gill (CPMG, RD—90°, (τ—180°) n—acquire) pulse sequence, was used to obtain ^1^H-NMR spectra. Using presaturation to inhibit water, spectra were captured at 27 °C. The following acquisition parameters were used: 8278.146 Hz spectral width, 0.126 Hz free induction decay (FID) resolution, 60.40 dwell time (DW), 1 s relaxation delay, 3.95 s acquisition time, and 16 scans. Sixteen signal averages (NSAs) were applied to a 90° pulse. Top-Spin 4.0.7 software was used to process the resulting spectra, including baseline and phase correction. Data were adjusted to the total integrated area after spectra ranging from 0 to 12 ppm were examined. Chemical changes were compared to those in human metabolite databases in order to identify metabolite signals.

The internal standard for chemical shift reference and measurement was trimethylsilyl propanoic acid (TSP). TSP was selected because of its single, potent signal at 0.00 ppm, which comes from its three methyl groups’ 14 equivalent protons. TSP is appropriate for this application since it is non-reactive and has a low boiling point.

### 2.5. Metabolite Identification and Spectral Analysis

By comparing the chemical shifts observed in the ^1^H-NMR spectra to those listed in the Human Metabolome Database (HMDB), metabolites were identified. The Bruker TopSpin 4.0.7 program was used to perform spectral analysis, which included peak picking, integration, and J-coupling analysis. Spin–spin coupling patterns, peak areas for quantification, and individual metabolite signals were identified and assigned using chemical shift values. By confirming that the chemical shift matched the HMDB within 0.01 ppm, each metabolite signal was accurately identified.

### 2.6. Data Processing

MestReNova software (version 12.0.0, MestreLab Research, Santiago de Compostela, Spain) was used to process and show H-NMR spectra. Metabolite concentrations were determined using quantitative NMR methods as described by Jaikang et al. [22]. Briefly, the concentration of each metabolite was determined by measuring its intensity and applying the following equation:

$$I_{A}=\frac{H_{A}\times C_{A}}{H_{B}\times C_{B}}\times I_{B}$$

where I_A_ represents the intensity of the metabolite, I_B_ is the intensity of TSP, H_A_ is the number of hydrogen atoms in the metabolite, and H_B_ corresponds to the number of hydrogen atoms in TSP (H_B_ = 14). Additionally, C_A_ denotes the metabolite concentration, while C_B_ refers to the TSP concentration (μm).

### 2.7. Statistical Analysis

The mean ± standard deviation (SD) is used to display the data. The normality of the data was evaluated using the Kolmogorov–Smirnov test. The Mann–Whitney U test was used to assess the differences in metabolite levels between the exposure and control groups. A *p*-value of less than 0.05 was considered statistically significant.

### 2.8. Metabolomics Data Analysis

A free online program for metabolomics data processing and visualization, MetaboAnalyst 6.0 (http://www.metaboanalyst.ca/MetaboAnalyst, accessed on 12 January 2024), was used to analyze the data. Outliers, missing values, and non-negative values were verified in the data before analysis, and they were eliminated. Following median normalization, the data underwent auto-scaling and log-transformation (base 10). To investigate the variations in metabolic profiles between the exposure and control groups, principal component analysis (PCA) was employed. Using 10-fold cross-validation and permutation testing, the model’s quality was evaluated by its goodness of fit (R2) and prediction (Q2). Significant metabolite changes were visualized using volcano plots; a fold change (FC) > 2 and a *p*-value < 0.05 were considered significant. The global test enrichment approach was utilized to analyze metabolic pathways, while relative betweenness centrality was employed to analyze pathway topology. The Homo sapiens (KEGG) database served as the foundation for the pathway mapping.

## 3. Results

### 3.1. Participant Demographic Information

One hundred and ninety-seven healthy participants were categorized into two groups: the exposure group (EG) (*n* = 99) and the control group (CG) (*n* = 98). The EG and CG groups comprised 72 and 71 females, respectively. Age, gender, weight, height, blood pressure, and heart rate were similar between the groups, and the results are presented in Table 1.

### 3.2. Analysis of Different Metabolites Between the Particulate Matter Exposure and the Control Group

**Figure 1:**
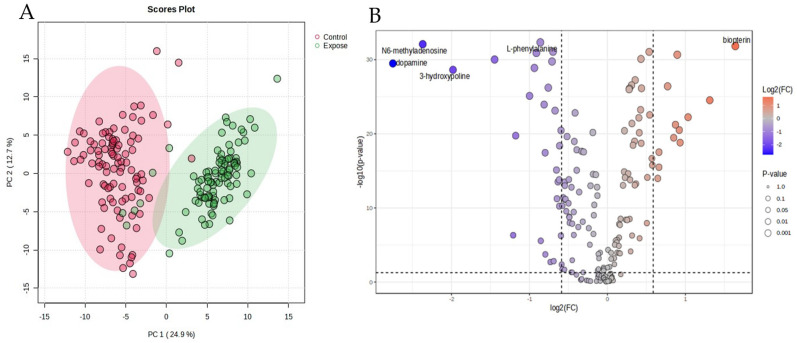
Principal component analysis (PCA, (**A**)) and volcano plot (**B**) of the particulate matter exposure group and the control group.

In the blood samples, two hundred and ten metabolites were identified by ^1^H-NMR. The metabolites were normalized by median, transformed by logarithms, and scaled by mean centering before analysis. The data of the chemometric analysis are shown as a principal component analysis (PCA) in Figure 1A. The PCA shows the difference in the blood metabolites between the EG and the CG groups. The figure demonstrates that the characteristics of the high- and low-exposure areas were separated with principal components 1 and 2, as 24.9 and 12.7%, respectively.

The variations in metabolite levels between the exposure and control groups are depicted by the volcano plot. Individual metabolite data points are displayed on the x-axis along with their fold changes (log2 FC) and statistical significances (−log10 *p*-value) on the y-axis. As shown by their placement in the plot’s upper-right quadrant, a total of 12 metabolites were markedly elevated in the control group when compared to the exposure group. A *p*-value threshold of *p* < 0.05 and a fold change cutoff of 1.5 or −1.5, indicated by dashed lines on the plot, were used to evaluate significance. These data are presented in Figure 1B. The important metabolites for which the fold change value in the PM exposure group was greater than in the control group are shown in Table 2. Every metabolite’s concentration in the exposure group contrasted with that of the control. Group differences in the 41 metabolites are displayed as volcano plots (Figure 1B). Dopamine, N6-methyladenosine, 3-hydroxyproline, 5-carboxylcytosine, and cytidine were all markedly elevated in the HG group as opposed to the CG. The CG displayed considerably higher levels of biopterin, adenosine, L-Histidine, epinephrine, and norepinephrine compared to the EG. Long-term exposure to PM2.5 degrades energy metabolism, amino acid metabolism, and increases oxidative stress, according to the metabolic tests.

### 3.3. Analysis of Long-Term PM2.5 Exposure-Related Diseases

**Figure 2:**
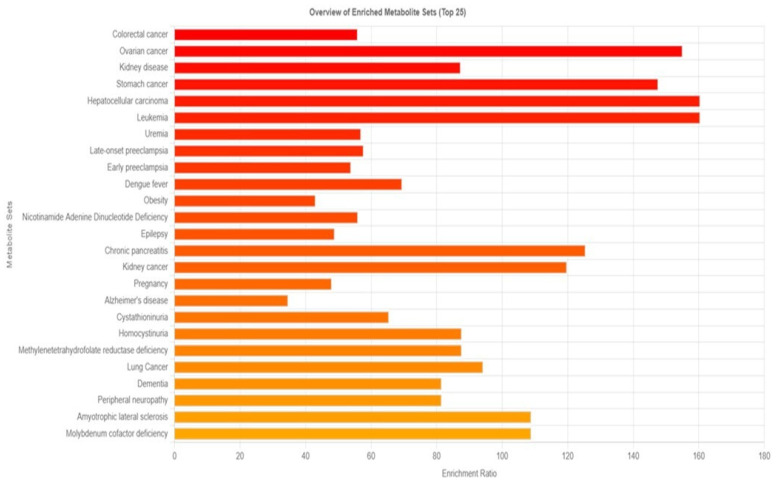
Overview of enrichment metabolite sets and possible diseases compared between the group with particulate matter exposure and the control group.

The disease signature in blood was identified using 210 metabolites, as opposed to the 480 sets reported in human blood in Metaboanalyst 6.0. The results were displayed in Figure 2, which suggested that 159 diseases might be triggered by prolonged exposure to PM2.5. Cancers, particularly those of the colon, ovaries, stomach, liver, and white blood cells, are the main illnesses linked to prolonged exposure to PM, according to enriched metabolite sets.

### 3.4. Impact of Particulate Exposure to Metabolic Pathways

**Figure 3:**
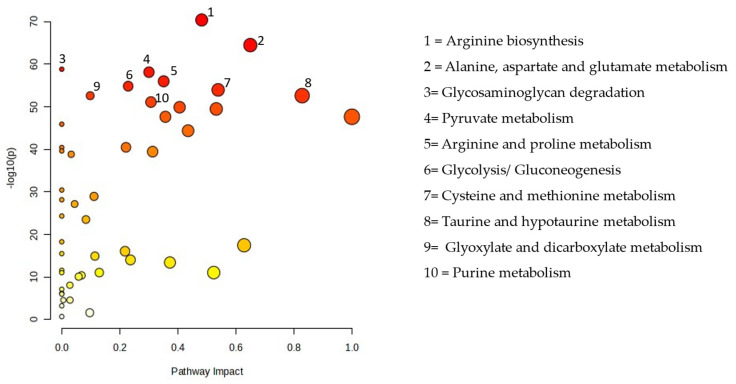
Pathway analysis of the particulate matter exposure group compared with the control group.

Figure 3 displays all matching routes based on the pathway effect values from the pathway topology analysis and the *p* values from the pathway enrichment analysis. PM exposure altered 48 pathways, including the metabolism of sugar, purines, lipids, and amino acids. The pathway impact between the EG and CG are presented in Table 3.

## 4. Discussion

Environmental pollution has risen alongside industrial development, posing a significant threat to human health. In 2013, the International Agency for Research on Cancer reported atmospheric particulate matter (PM) as a Group 1 carcinogen, thereby recognizing it as a major contributor to mortality from respiratory and cardiovascular conditions and a significant global environmental risk factor [30]. PM is a complex mixture of metal, chemical, and organic substances that are extremely reactive. It primarily affects health by causing the excessive formation of reactive oxygen species (ROS), which can lead to endoplasmic reticulum stress, inflammatory reactions, atherosclerosis, airway remodeling, and damage to DNA and cells. These effects increase vulnerability and exacerbate various diseases and infections. Air pollution in Thailand is largely driven by fine particulate matter, with seasonal variations and differing patterns across regions [31]. Chiang Mai, the largest city in Northern Thailand, situated on a plain surrounded by high mountains, experiences a haze season that begins in January and ends in April. During this period, Chiang Mai often ranks among the top ten cities globally for dust levels contributing to air pollution, the primary sources of PM2.5 and PM10 during haze season are biomass burning from agricultural activities and wildfires. In contrast, Sa Kaeo, an eastern Thai city located on a plain near the sea, also faces particulate matter pollution from agricultural biomass burning but at lower levels compared to Chiang Mai. The mean PM2.5 concentrations in high- and low-exposure areas were 31.7 µg/m^3^/day and 19.5 µg/m^3^/day, respectively, while PM10 levels averaged 42.3 µg/m^3^/day in high-exposure areas and 32.8 µg/m^3^/day in low-exposure areas. Compared to low-exposure areas, PM2.5 and PM10 concentrations were approximately 1.6 and 1.3 times higher in high-exposure areas, respectively. Notably, the average levels of both pollutants in both areas exceeded the World Health Organization (WHO) air quality guideline thresholds [32]. PM2.5 and PM10 concentrations were monitored by the Thai Meteorological Department from June 2014 to December 2023. In high-exposure areas, PM2.5 levels varied between 23 and 36 µg/m^3^/day, while PM10 ranged from 32 to 59 µg/m^3^/day. In contrast, low-exposure areas exhibited lower ranges, with PM2.5 levels between 17 and 25 µg/m^3^/day and PM10 levels between 9 and 45 µg/m^3^/day.

Our findings suggest that prolonged exposure to PM2.5 disrupts energy metabolism and amino acid metabolism while also increasing oxidative stress. Engaging in strenuous activities during periods of high air pollution can elevate energy production due to increased physical exertion, leading to greater oxygen consumption and metabolic demand. However, exposure to fine particulate matter (PM2.5) has been associated with impaired glucose and insulin tolerance, as well as decreased energy expenditure [33]. We observed lower concentrations of succinate, citrate, and malate, indicating the suppression of the tricarboxylic acid (TCA) cycle. This disruption likely hinders electron transfer to ubiquinone, thereby impairing oxidative phosphorylation and reducing ATP production [34].

Mitochondria are highly susceptible to external stressors and serve as primary targets for PM2.5-induced cellular damage. The resulting disruption of mitochondrial structure and function contributes to the onset of various human diseases [35]. Alterations in mitochondrial dynamics, mitochondrial permeability transition pore activity, mitochondrial DNA, and oxidative stress occurred after PM2.5 exposure [36]. PM2.5 reduced the mitochondrial membrane potential (ΔΨm) and promoted the opening of the mitochondrial permeability transition pore, ultimately leading to disruptions in adenosine triphosphate (ATP) synthesis. These findings clearly demonstrate that PM2.5 induces endothelial toxicity by targeting mitochondria, resulting in the disruption of mitochondrial homeostasis [37]. PM2.5 exposure led to mitochondrial structural damage, including disruptions in mitochondrial dynamics, DNA biogenesis, and morphological alterations in 16HBE cells. Additionally, PM2.5 increased reactive oxygen species production [38]. Metabolomics is a valuable tool for assessing mitochondrial function, with various approaches requiring different levels of resource investment for data acquisition and analysis. Targeted metabolomics, which focuses on a specific set of analytes, is recommended as an initial strategy. An analysis of intermediates of the TCA cycle, glutaminolysis, and glycolysis can provide metabolomic workflow, highlighting the effectiveness of targeted metabolomics in mitochondrial research [39]. Decreases in basal respiration, maximal respiration, spare capacity, proton leak, and ATP production indicate that PM2.5 exposure leads to mitochondrial damage and disrupts cellular energy production. These results are consistent with previous studies highlighting mitochondrial dysfunction and energy metabolism impairments associated with PM2.5 exposure [40]. A study on GC-2spd cells demonstrated that PM2.5 exposure significantly decreased these mitochondrial parameters, highlighting mitochondrial dysfunction [21]. Similarly, research on BV2 microglial-like cells showed that urban particulate matter (UPM) exposure led to the rapid impairment of mitochondrial function and increased mitochondrial fragmentation, further supporting the notion that particulate matter exposure adversely affects mitochondrial health [41].

Impaired energy metabolism can hinder the active transport of essential nutrients, such as amino acids and nucleotides, potentially disrupting biological processes and affecting cell proliferation [21]. Prolonged exposure to PM2.5 also altered the kynurenine pathway, as indicated by elevated levels of 3-hydroxyanthranilic acid and hydroxykynurenine. Additionally, xanthurenic acid and kynurenic acid—key metabolites in tryptophan metabolism—were upregulated, possibly due to the activation of tryptophan 2,3-dioxygenase (TPHD) and modifications in the kynurenine pathway [42]. The dysregulation of this pathway has been linked to several central nervous system disorders, including depression, epilepsy, and schizophrenia [43,44].

To assess the potential health risks linked to the observed metabolic disruptions, enrichment analysis was performed using MetaboAnalyst 6.0. By comparing our 210 identified blood metabolites with a reference database of 480 metabolites, we identified 159 diseases potentially influenced by long-term PM2.5 exposure (Figure 2). Among these, cancer emerged as the most significant disease category, with notable associations with colon, ovarian, kidney, stomach, liver, blood, and lung cancers. This finding is consistent with previous research demonstrating an increased cancer risk associated with PM2.5 exposure. For example, a meta-analysis reported a significant correlation between PM2.5 exposure and colorectal cancer (CRC) risk, with an overall risk of 1.19 (95% CI: 1.12–1.28). Regional variations were observed, with North America exhibiting higher incidence and mortality rates than Asia. In the United States, specifically, the odds ratios for incidence and mortality were 1.61 (95% CI: 1.38–1.89) and 1.29 (95% CI: 1.17–1.42), respectively [45]. Another study found that a 10 μg/m^3^ increase in black carbon, organic carbon, and dust-PM2.5 levels corresponded to a 4%, 4%, and 15% increase in colon cancer risk, respectively [46]. Beyond cancer, our analysis also identified neurodegenerative diseases, including Alzheimer’s disease (AD) and dementia, as potential health risks associated with prolonged PM2.5 exposure. PM2.5 has been linked to neuroinflammation, oxidative stress, and epigenetic modifications, all of which may contribute to the development of AD and other neurological disorders [47].

To further explore the biological effects of PM2.5 exposure, we conducted pathway analysis using MetaboAnalyst 6.0. This analysis identified 45 metabolic pathways significantly affected by PM2.5 exposure, including those related to amino acid, lipid, purine, and sugar metabolism (Figure 3). Among these, glyoxylate and dicarboxylate metabolism exhibited the most substantial disruption. These findings align with previous research demonstrating that PM2.5 exposure alters various metabolic pathways in the lungs. A study in mice revealed that PM2.5 exposure modified lung metabolites associated with the citrate cycle; glyoxylate and dicarboxylate metabolism; pyruvate metabolism; purine and pyrimidine metabolism; and valine, leucine, and isoleucine metabolism. Additionally, changes in the lung microbiota were linked to alterations in lung metabolites, suggesting a complex relationship between PM2.5 exposure, metabolic disturbances, and the lung microbiome [36]. These results further support the idea that PM2.5 exposure not only disrupts metabolic pathways but also affects the composition of the lung microbiome, ultimately contributing to lung injury.

Our study also highlights the disruption of glutathione metabolism due to PM2.5 exposure. Glutathione plays a vital role in defending cells against oxidative stress by neutralizing reactive oxygen species. Nassan et al. identified several metabolic pathways affected by PM2.5 exposure, including glycerophospholipid, sphingolipid, and glutathione metabolism, which are involved in inflammation, oxidative stress, immune responses, and nucleic acid damage repair [13]. Additionally, Wang et al. demonstrated that glutathione depletion, along with reduced activity of glutathione peroxidase and NADPH, contributes to PM2.5-induced endothelial cell ferroptosis, a form of cell death driven by iron-dependent lipid peroxidation [48].

There are some limitations in this study, including the fact that PM2.5 and PM10 levels were averaged over specific locations but individual exposure levels may vary due to indoor air pollution, occupational exposure, personal protective behaviors (e.g., mask usage), and environmental or lifestyle factors (e.g., diet, smoking, and genetics). Further validation with targeted metabolomics and larger population-based studies would strengthen these findings.

## 5. Conclusions

This cross-sectional study examined the health effects of prolonged PM2.5 exposure by comparing individuals from high- and low-exposure areas in Thailand. Our results indicate that chronic PM2.5 exposure disrupts key metabolic pathways, contributing to increased oxidative stress, mitochondrial dysfunction, and impairments in energy and amino acid metabolism. These metabolic disturbances are linked to a heightened risk of various diseases, including cancer and neurodegenerative disorders. Further research is essential to better understand the long-term health impacts of PM2.5 exposure in under-represented populations and regions. Future studies should also aim to improve exposure assessment methodologies and investigate the relationship between PM2.5 exposure and mortality from specific diseases, such as diabetes mellitus, kidney disease, dementia, and lung cancer.

## Figures and Tables

**Table 1:** Demographic data of the participants.

Parameters	Exposure (*n* = 99)	Control (*n* = 98)	*p*-Value
Sex: Female	72 (72.7%)	71 (72.4%)	0.218
Age (year)	46.9 ± 12.3	41.7 ± 13.8	0.080
Weight (kg)	62.3 ± 12.1	64.8 ± 12.6	0.272
Height (cm)	159.1 ± 12.0	161.6 ± 8.6	0.971
Body Mass Index, BMI (kg/m^2^)	23.36 ± 4.0	24.5 ± 3.9	0.443
Systolic Pressure (mmHg)	130.1 ± 16.4	129.7 ± 22.9	0.200
Diastolic Pressure (mmHg)	81.2 ± 9.9	82.1 ± 14.5	0.397
Heart Rate (beat/min)	81.9 ± 11.7	82.1 ± 14.7	0.601

Data are expressed as mean ± standard deviation. The data were compared with an independent *t*-test.

**Table 2:** The significant metabolites in the particulate matter exposure group compared with the control groups.

Metabolites	Fold Change	log2 (FC)	−log10 (*p*)
Dopamine	0.15	−2.75	29.49
N6-methyladenosine	0.19	−2.36	32.08
3-Hydroxyproline	0.25	−1.98	28.62
5-Carboxylcytosine	0.37	−1.44	30.02
Cytidine	0.43	−1.20	6.31
Betaine	0.44	−1.17	19.74
5-Hydroxylysine	0.50	−0.99	25.11
4-Hydroxyproline	0.52	−0.94	28.88
L-Arginine	0.53	−0.91	30.91
L-Phenylalanine	0.55	−0.86	32.33
1-Methylnicotinamide	0.55	−0.85	5.55
5-Formylcytosine	0.57	−0.81	23.94
Ornithine	0.57	−0.79	17.44
Dihydrofolate (7,8-dihydrofolate)	0.58	−0.79	3.73
Homocysteine	0.59	−0.75	26.22
1-Methyladenosine	0.60	−0.73	2.96

**Table 3:** Pathways impact analysis between the particulate matter exposure group compared with the control group.

Pathways	Match Status	−log (*p*)	Holm *p*	Impact
Arginine biosynthesis	7/14	70.40	1.96 × 10^−69^	0.48
Alanine, aspartate, and glutamate metabolism	11/28	64.49	1.57 × 10^−63^	0.65
Glycosaminoglycan degradation	1/23	58.83	6.95 × 10^−58^	0.0
Pyruvate metabolism	7/23	58.13	3.37 × 10^−57^	0.30
Arginine and proline metabolism	8/36	55.99	4.53 × 10^−55^	0.35
Glycolysis/Gluconeogenesis	5/26	54.84	6.31 × 10^−54^	0.23
Cysteine and methionine metabolism	8/33	53.97	4.56 × 10^−53^	0.53
Taurine and hypotaurine metabolism	3/8	52.62	1.00 × 10^−51^	0.83
Glyoxylate and dicarboxylate metabolism	10/32	52.61	1.01 × 10^−51^	0.10
Purine metabolism	15/70	51.11	3.06 × 10^−50^	0.31
Citrate cycle (TCA cycle)	8/20	49.89	4.91 × 10^−49^	0.41
Tyrosine metabolism	10/42	49.95	1.22 × 10^−48^	0.41
Phenylalanine, tyrosine, and tryptophan biosynthesis	2/4	47.65	8.16 × 10^−47^	1.0
Phenylalanine metabolism	2/8	47.66	8.16 × 10^−47^	0.35
Pantothenate and CoA biosynthesis	4/20	45.91	4.22 × 10^−45^	0.0
Glycine, serine, and threonine metabolism	10/33	44.37	1.42 × 10^−43^	0.43
Histidine metabolism	4/16	40.48	1.08 × 10^−39^	0.22
Thiamine metabolism	1/7	40.43	1.18 × 10^−39^	0.0
D-amino and metabolism	2/15	39.62	7.35 × 10^−39^	0.0
Glutathione metabolism	7/28	39.46	1.03 × 10^−38^	0.31

## Data Availability

The original contributions presented in the study are included in the article, and further inquiries can be directed to the corresponding author.
